# Increased Threat of Thyroid Diseases in Patients With Sjogren’s Syndrome: A Systematic Review

**DOI:** 10.7759/cureus.28062

**Published:** 2022-08-16

**Authors:** Harkirat Kaur, Mohammad Alazzeh, Abhay Thandavaram, Aneeta Channar, Ansh Purohit, Bijay Shrestha, Deepkumar Patel, Hriday Shah, Kerollos Hanna, Lubna Mohammed

**Affiliations:** 1 Family Medicine, California Institute of Behavioral Neurosciences & Psychology, Fairfield, USA; 2 Orthopedic Surgery, California Institute of Behavioral Neurosciences & Psychology, Fairfield, USA; 3 Internal Medicine, California Institute of Behavioral Neurosciences & Psychology, Fairfield, USA; 4 Research, California Institute of Behavioral Neurosciences & Psychology, Fairfield, USA; 5 Neurology, California Institute of Behavioral Neurosciences & Psychology, Fairfield, USA

**Keywords:** exocrine glands, thyroid gland, women’s health, anti-ssa antibody, internal medicine, autoimmune disoders, endocrinology, autoimmune thyroid disorder, thyroid disorder, sjogren's syndrome

## Abstract

Sjogren's syndrome is an autoimmune disorder of the body's exocrine glands; however, it is known to have numerous extra-glandular and endocrine manifestations in the body. Moreover, other autoimmune have also been reported with high prevalence in patients with Sjogren's syndrome, including thyroid diseases. Therefore in this study, we aimed to ascertain the increased risk of developing thyroid disorders in patients with pre-existing Sjogren's syndrome. The systematic review was conducted according to the Preferred Reporting Items for Systematic Reviews and Meta-Analyses (PRISMA) guidelines. Online searches on PubMed, PubMed Central (PMC), Google Scholar, and Cochrane were done till 5th June 2022 to filter out studies published in the last twenty years. Based on the inclusion-exclusion criteria, 167 studies were initially selected. They were screened and assessed by quality assessment tools that yielded seven studies, including one meta-analysis, three non-randomized control trials, and three systematic reviews. The study proved that patients with Sjogren's syndrome are at significant risk of developing thyroid disorders, especially autoimmune thyroiditis. This also highlights the need for advanced research and better diagnostic and screening protocols for these patients to reduce the seriousness of the disease.

## Introduction and background

Epidemiological statistics suggest that an estimated world population of 0.5-1% have Sjogren's syndrome [1]. This syndrome, which is also known as Sicca syndrome, is an autoimmune disease where the cells of a patient's own body tend to attack its exocrine glands. The lacrimal and salivary glands are severely damaged in these patients, leading to grittiness in the eyes and dry mouth respectively. With a female-to-male ratio of 9:1, this disease primarily affects middle-aged women [2].

Henrik Sjorgren was the first scientist in the early twentieth century to have identified the prevalence of these symptoms among a group of female patients who were also known to have polyarthritis [3]. We now know that lymphocytic infiltration of salivary and lacrimal glands is the highlight of this syndrome. However, due to incomplete elucidation of the pathogenetic pathways underlying Sjogren's disease, there is a paucity of pathophysiological knowledge about this autoimmune exocrinopathy.

Several extra glandular manifestations in the body have been noted in Sjogren's syndrome, leading to a spectrum of symptoms like fatigue, dry skin, cough due to xerotrachea, esophageal dysmotility, peripheral neuropathy, and thyroid diseases [4]. The American-European Consensus Group (AECG) has therefore put forward an extensive diagnostic method for Sjogren's syndrome, including criteria like ocular symptoms, oral symptoms, ocular signs, histopathological study, oral signs, and levels of autoantibodies [5]. The syndrome can be seen as an entity on its own, that is, as primary Sjogren's disease (pSS), or in association with other autoimmune disorders like rheumatoid arthritis or systemic lupus erythematosus (SLE), which is known as secondary Sjogren's syndrome, which can also be assessed by the AECG criteria. Therefore it is common to see a diverse range of autoantibodies like anti-Sjögren's-syndrome type A (anti-SSA/Ro) and anti-Sjögren's-syndrome type B (anti-SSB/La) autoantibodies, anti-nuclear antibodies (ANA), rheumatoid factor (RF) and even cryoglobulins during diagnostic analysis [6].

This also raises the question of the association of other autoimmune disorders in these patients. The interrelationship of SLE and rheumatoid arthritis with Sjogren's syndrome has been well established by extensive studies; however, in the past few decades, there has also been an increment in the reports of thyroid disorders in patients of Sjogren's syndrome [6-8]. This systematic review aims to analyze the risk of the same in these patients, which may assist in avoiding complications and improving outcomes and severity of Sjogren's syndrome.

## Review

Methods

This systematic research aims to identify the presence of thyroid diseases, especially autoimmune thyroiditis, in patients with pre-existing Sjogren's syndrome. This systematic review has been conducted according to the Preferred Reporting Items for Systematic Reviews and Meta-Analyses (PRISMA) 2020 guidelines [9].

Eligibility Criteria 

The studies that were selected are based on the participants and outcomes as follows:

Participants: Selection of studies with young adult and middle-aged group individuals (age 20-60 years) from all ethnicities and genders with Sjogren's syndrome or Sicca syndrome with or without other diagnosed autoimmune diseases.

Outcomes: Studies showing evidence of concurrent thyroid diseases, hypothyroidism, or hyperthyroidism in these patients have been selected.

Inclusion and Exclusion Criteria

In addition, inclusion and exclusion criteria were added as follows: papers of all languages with English translations, free full-text articles published within the past 20 years, randomized controlled trials (RCTs), non-randomized control trials, meta-analyses, literature and systematic reviews were included in the study. Case studies, case reports, animal studies, and editorials were excluded from the study. 

Search Strategy

The search was conducted online on PubMed, PubMed Central (PMC), Google Scholar, and Cochrane. The last search on all databases was on June 5th, 2022. Keywords presented in the literature were targeted by Medical Subject Headings search (MeSH), including Boolean operators and free text word searches to assure appropriate results from journals. "Sjogren's syndrome" or "Sicca syndrome" as well as "thyroid diseases", "thyroiditis", "hypothyroidism" or "hyperthyroidism" have been used in most of the journal search engines. Different search strategies used in various journals have been summarized in Table 1.

**Table 1:** A summary of search strategies used in PubMed, PubMed Central, Google Scholar, and Cochrane

Database	Keywords	Search strategy	Filters used	Results
PubMed	Sjogren's syndrome, Sicca syndrome, thyroid disorders, hypothyroidism, hyperthyroidism	(( "Sjogren's Syndrome/diagnosis"[Mesh] OR "Sjogren's Syndrome/epidemiology"[Mesh] OR "Sjogren's Syndrome/etiology"[Mesh] OR "Sjogren's Syndrome/genetics"[Mesh] OR "Sjogren's Syndrome/history"[Mesh] OR "Sjogren's Syndrome/immunology"[Mesh] OR "Sjogren's Syndrome/statistics and numerical data"[Mesh] )) AND ( "Thyroid Diseases/analysis"[Mesh] OR "Thyroid Diseases/complications"[Mesh] OR "Thyroid Diseases/epidemiology"[Mesh] OR "Thyroid Diseases/etiology"[Mesh] OR "Thyroid Diseases/genetics"[Mesh] OR "Thyroid Diseases/history"[Mesh] OR "Thyroid Diseases/immunology"[Mesh] OR "Thyroid Diseases/physiopathology"[Mesh] OR "Thyroid Diseases/prevention and control"[Mesh] OR "Thyroid Diseases/statistics and numerical data"[Mesh] ) - 297	Published between 2002-2022	125
PubMed Central	Sjogren's syndrome, thyroid disorders	"Sjogren's syndrome"[MeSH Major Topic] AND "thyroid diseases"[MeSH Major Topic] - 17	No filters used	17
Google Scholar	Sjogren's syndrome, Sicca syndrome, thyroiditis, thyroid disorders, hypothyroidism, hyperthyroidism	Sjogren's syndrome and thyroid disorders - 12,000	Published between 2002-2022	26
Cochrane	Sjogren's syndrome, thyroid disease	Sjogren's syndrome and thyroid disease - 10	No filters used	1

Risk of Bias in Included Studies

Two authors investigated the risk of bias using tools like the Scale for the Assessment of Narrative Review Articles (SANRA) and the Assessment of Multiple Systematic Reviews (AMSTAR) 2 checklist [10, 11]. Each study was assessed by these tools and scored accordingly. Studies with a minimum accepted scoring of >70% in SANRA and AMSTAR 2 checklists were selected. This selection has been briefly summarized in Table 2.

**Table 2:** Quality assessment tools used in the review and their scoring characteristics for selection criteria SANRA: Scale for the Assessment of Narrative Review Articles [10]; AMSTAR: Assessment of Multiple Systematic Reviews [11]; PICO: Population, Intervention, Comparison, Outcome

Quality assessment tool	Study type	Characteristics of assessment tool	Total score	Accepted score (>70%)	Accepted studies for this review
SANRA checklist	Narrative review articles	6 items: the importance of the article for readership, statement of concrete aims of formulation of questions, literature search description, referencing, scientific reasoning, presentation of data	12	9	4: Baldini et al. 2018 [12]; Tincani et al. 2013 [13]; Lazarus et al. 2005 [14]; Kassan et al. 2004 [15]
AMSTAR 2 checklist	Systematic reviews	16 items: inclusion of PICO, establishment of review methods prior to conducting the review and reports of deviations, explanation of study selections included in review, use of comprehensive search strategy, study selection in duplicate, extraction of data in duplicate, excluded studies and reason for exclusion, adequately detailed description of included studies, risk of bias included in the study, inclusion of funding sources, statistical combination of results in meta-analysis, impact of risk of bias by studies in the meta-analysis if included, risk of bias in the results of review, heterogeneity observed in the results of review, publication bias assessment, reports of conflict of interest	16	12	3 : Sun et al. 2019 [16]; Lu et al. 2013 [17]; Dai et al. 2021 [18]

Results

**Figure 1:**
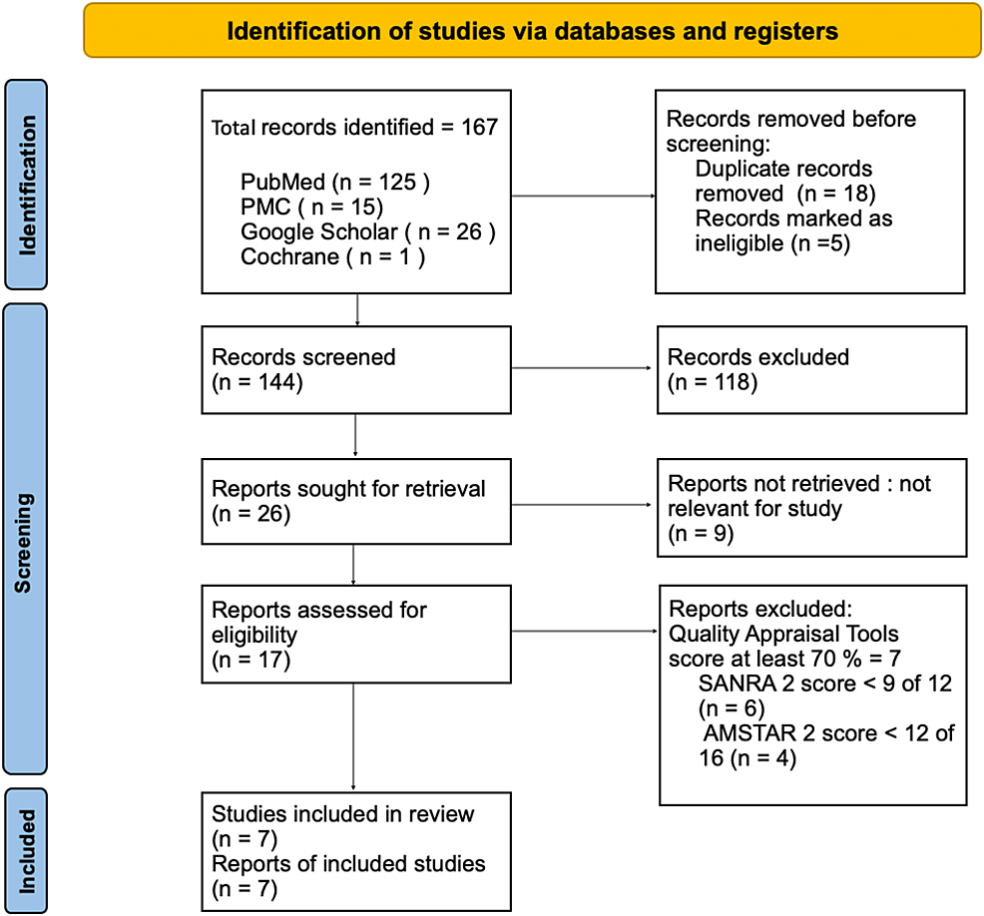
PRISMA flow diagram for the study PRISMA: Preferred Reporting Items for Systematic Reviews and Meta-analyses [9]; PMC: PubMed Central; SANRA: Scale for the Assessment of Narrative Review Articles [10];  AMSTAR: Assessment of Multiple Systematic Reviews [11]

The search on databases resulted in 167 titles that were relevant to this systematic review. These titles were managed on reference management software EndNote (Clarivate, London, United Kingdom), and 18 duplicate records were removed along with five ineligible titles. A total of 147 records were screened out of which 26 titles were sought for retrieval. Seventeen records were singled out according to eligibility criteria and were assessed by quality appraisal tools with a score of a minimum of 70%. Ultimately seven records were selected for this systematic review, including one systematic review and meta-analysis, three retrospective studies, and three literature reviews. The database identification and screening can be summarized by the PRISMA chart depicted in Figure 1.

Study Characteristics

Table 3 lists the studies that were selected for this review according to the inclusion criteria, along with their titles, publishing years, and the type of study that was included. It briefly describes the outcome of the respective studies as well.

**Table 3:** A list of the seven studies that were selected in this systematic review after screening SS: Sjogren's syndrome, AITD: autoimmune thyroid disorder

Author	Name of study	Publishing year	Type of study included in the review	Outcome of study
Baldini et al. [12]	The association of Sjogren's syndrome and autoimmune thyroid disorders	2018	Literature review	The coexistence of SS and AITD is prevalent in clinical practice, most likely due to overlapping pathogenetic processes between the two diseases.
Tincani et al. [13]	Novel aspects of Sjogren's syndrome in 2012	2013	Literature review	Extraglandular symptoms continue to be a problem in the treatment of Sjogren's which requires more research
Lazarus et al. [14]	Development of additional autoimmune diseases in a population of patients with primary Sjogren's syndrome	2005	Retrospective study	The most frequent autoimmune illness was hypothyroidism, which was found in 16 cases out of 114 (14%)
Kassan et al. [15]	Clinical manifestations and early diagnosis of Sjogren's syndrome	2004	Literature review	The adoption of updated diagnostic criteria can aid in the early identification of people with SS
Sun et al. [16]	Increased risk of thyroid disease in patients with Sjogren's syndrome: a systematic review and meta-analysis	2019	Systematic review and meta-analysis	Thyroid disease was shown to be more common in SS patients than in controls, suggesting that SS patients should be checked for thyroid disease
Lu et al. [17]	Increased risk of primary Sjogren's syndrome in female patients with thyroid disorders: a longitudinal population-based study in Taiwan	2013	Retrospective study	A considerable rise in the incidence of SS among female thyroid disease patients, especially those in their mid-forties to mid-sixties
Dai et al. [18]	Clinical features and laboratory examination results of Sjogren's syndrome complicated with thyroid disorders: a retrospective analysis	2021	Retrospective study	SS is frequently accompanied by hypothyroidism, and it has a physiological and pathological basis with autoimmune thyroid disorders

Discussion

This section of the review discusses the prevalence of thyroid disorders, especially autoimmune thyroiditis, in patients with Sjogren's syndrome and the similar pathogenesis of both these autoimmune disorders. Apart from the above, the antibodies responsible for their pathogenesis and the role of early screening of thyroid dysfunction in these patients will also be discussed.

Prevalence of Thyroid Disorders in Sjogren's Syndrome

Sjogren's syndrome is an autoimmune disorder known to have multifarious manifestations in the human body. Apart from dry eyes and mouth, an association of respiratory, dermatological, gastrointestinal, and neurological disorders has also been extensively studied [13, 15]. Numerous studies in the past have already established the risk of these patients having other autoimmune disorders like systemic lupus erythematosus (SLE).

However, the prevalence of thyroid disorders has also been increasingly documented and studied due to better screening practices globally. As per the American-European Consensus Group (AECG) criteria for diagnosis, patients with pre-existing Sjogren's syndrome have been chosen in several studies to demonstrate the same. Most studies have documented an increased risk of autoimmune thyroid disease or hypothyroidism instead of hyperthyroidism in patients with Sjogren's. In a study published in 2021, out of a group of 201 patients with Sjogren's syndrome, a significant number had greater positive rates for antithyroglobulin antibody (aTG) and thyroid peroxidase antibodies (aTPO) than patients in the normal group in a study published in 2021 [18]. This confirmed the presence of thyroid disorders, especially hypothyroidism, in this study group.

A retrospective study from 2005 was conducted with 114 patients of Sjogren's syndrome, who were studied for other autoimmune disorders. It was concluded that 14% of patients have an association with hypothyroidism [14]. Similarly, a retrospective study from Taiwan included 389 female patients with Sjogren's that concluded with an increased documented risk of hypothyroidism in them [17]. A meta-analysis that sought to establish a risk of thyroid disorders in Sjogren's syndrome in 2019 concluded with significant results. The study showed that the risk of autoimmune thyroiditis was increased in these patients as compared to controls [16].

Most of the studies have documented an increased risk of autoimmune thyroid disease or hypothyroidism instead of hyperthyroidism in patients with Sjogren's.

Similar Pathogenesis

It has been well established that the pathogenesis of Sjogren's syndrome includes lymphocytic infiltration of the body's exocrine glands, a process that is brought about by the glandular epithelial cells [15]. A genetically susceptible patient with the presence of auto-antibodies is predisposed to the development of glandular dysfunction caused by this syndrome.

**Figure 2:**
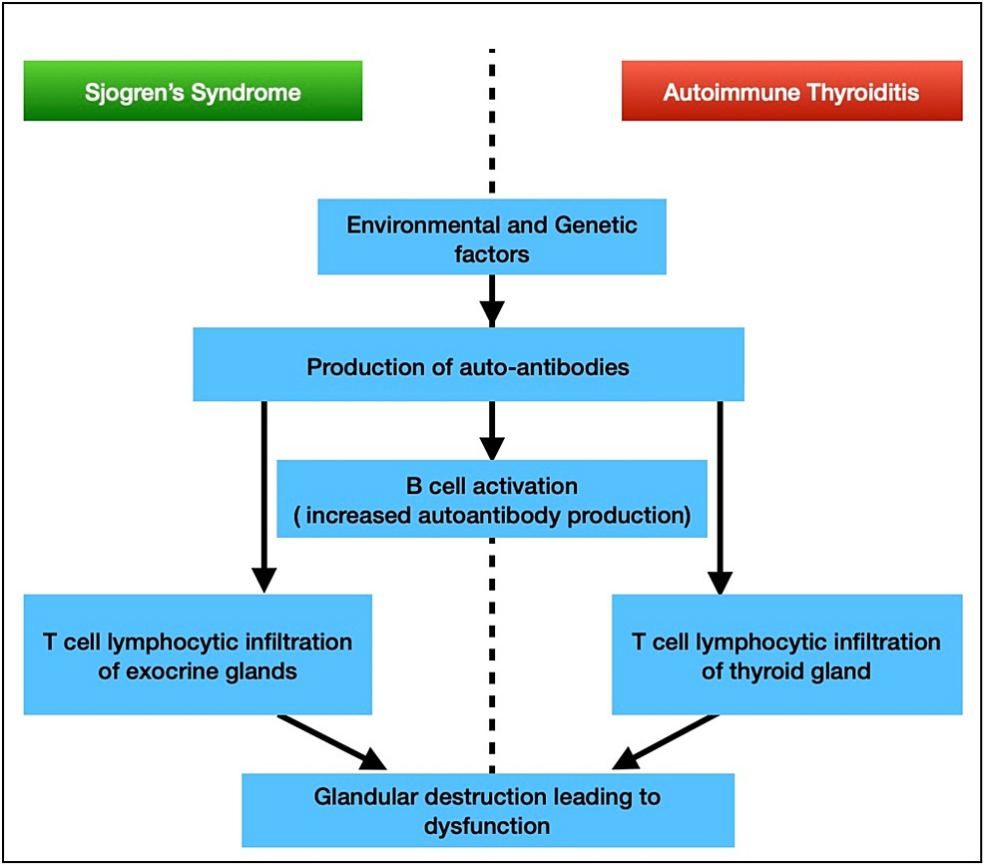
A similarity between the pathogenesis of Sjogren's syndrome and autoimmune thyroiditis has been briefly explained by this flowchart T cell lymphocytic infiltration of the glands in both these autoimmune diseases is a hallmark feature which leads to dysfunction

The cascade of inflammatory glandular insult seen in Sjorgen's is similar to the pathophysiology seen in autoimmune thyroid disease. These similarities can be discussed by several noteworthy points. For example, CD4+ T cells are known to be predominant in the gland infiltrate in both these autoimmune disorders. The activation of B cells in the pathogenesis is now known to play a key role in the progression of these diseases as well. A summary of the identical pathogenesis of Sjogren's syndrome and autoimmune thyroid disease can be seen in Figure 2.

Moreover, the expression of human leukocyte antigens (HLA) like HLA-DR3 and HLA-B8 is significant in the populations with Sjogren's and autoimmune thyroid disease [12]. The role of chemokines like CXCL10 has also been documented in glandular destruction in these pathologies [19].

Screening Practices

The AECG criteria revised in 2012 for the diagnosis of Sjogren's syndrome include the presence of any two of the three points: anti-Sjogren's syndrome type A antibody (Anti-SSA) and/or anti-Sjogren's syndrome type B antibody (Anti-SSB) levels; an ocular staining score of ≥3; salivary gland biopsy showing lymphocytic sialadenitis [5].

However, with evidence of an increased risk of hypothyroidism in patients with Sjogren's syndrome, the importance of screening practices that can help rule out the same is rising too. Patients with a positive family history of thyroid disorders are genetically susceptible due to the possibility of circulating auto-antibodies in their blood. Therefore, thyroid stimulating hormone (TSH) levels, aTg and thyroid peroxidase (TPO) antibody testing is crucial in establishing the diagnosis of hypothyroidism or for evaluating risk. This was seen in a study published in 2021, where 74 patients out of 201 with Sjogren's had their thyroid function tests evaluated to diagnose a concomitant thyroid disorder [18].

Limitations

This study is limited because of the lack of randomized controlled trials related to thyroid disorders accompanying a diagnosis of Sjogren's syndrome. The study is predominantly concerned with the prevalence and association of both these disorders and therefore does not concentrate on a therapeutic approach. Imaging technology or biopsies for diagnosis have also not been discussed. There is also less data available regarding undiagnosed cases of thyroid disorders or patients with predisposed risk due to family history.

Lifestyle variations among patients, such as the history of smoking, drug use, alcoholism, or presence of other concomitant autoimmune disorders, have also not been explored. Additionally, the timelines for selected studies are spread across decades as autoimmune disorders have a variable progression. Follow-up of patients diagnosed with thyroid disorders had not been focused on for the same reason.

## Conclusions

Autoimmune disorders like Sjogren's syndrome are known to have a constellation of manifestations in the human body. The studies that have been included in this review have conclusively demonstrated a higher probability of the development of thyroid dysfunction in patients diagnosed with Sjogren's syndrome.

To mitigate the severity of Sjogren's syndrome and to enhance the quality of life of individuals with this disease who are genetically predisposed, physicians must explore the importance of improved screening protocols in patients showing extra-glandular symptoms. This will also open gates for improved research and better therapeutic protocols for these patients. Furthermore, as thyroid disorders and Sjogren's syndrome have predominantly female patients, this review explores their arena of improved healthcare practices.
